# Traditional Masculinity and Aggression in Adolescence: Its Relationship with Emotional Processes

**DOI:** 10.3390/ijerph18189802

**Published:** 2021-09-17

**Authors:** Elisabeth Malonda-Vidal, Paula Samper-García, Anna Llorca-Mestre, Roger Muñoz-Navarro, Vicenta Mestre-Escrivá

**Affiliations:** 1 Department of Basic Psychology, Faculty of Psychology, University of Valencia, 46010 Valencia, Spain; paula.samper@uv.es (P.S.-G.); anna.llorca@uv.es (A.L.-M.); maria.v.mestre@uv.es (V.M.-E.); 2 Department of Personality, Evaluation and Psychological Treatments, Faculty of Psychology, University of Valencia, 46010 Valencia, Spain; roger.munoz@uv.es

**Keywords:** reactive aggression, proactive aggression, masculinity, femininity, regulatory emotional self-efficacy, emotion regulations, adolescence

## Abstract

Traditional masculinity includes norms that encourage many of the aggressive behaviors whereas traditional femininity emphasizes aggression very little. In addition, the lack of emotional regulation as well as a poor impulse control have been related to aggression and, in particular, with reactive and proactive aggression. The objective of this study is to examine the role of gender stereotypes (masculinity/femininity) in reactive and proactive aggression, through regulatory emotional self-efficacy and emotion regulation. A total of 390 adolescents participated in a longitudinal study in Valencia, Spain. Structural equations modeling (SEM) was employed to explore a two-wave longitudinal model. The results show that femininity relates to reactive aggression through regulatory emotional self-efficacy and emotion regulation. This way, both emotional self-efficacy and emotional regulation mediate the relation between femininity and reactive aggression. Furthermore, reactive and proactive aggression relate positively and directly to masculinity and negatively to femininity. Therefore, violence prevention programs with adolescents should incorporate information to break down gender stereotypes and promote strategies to manage emotions. Such efforts may be helpful to reduce aggressive behaviors and violence.

## 1. Introduction

Aggression is defined as ‘behavior with the intent to harm oneself or others’, and is a common behavioral problem in adolescence [1]. Different types of aggressive behavior exist (e.g., physical, verbal, direct, indirect, reactive, proactive…), which are usually associated with other social functioning problems (crime, school bullying, intimate partner violence, or gender violence) [2,3,4]. However, in this study, we will focus on reactive and proactive aggression, their classification made according to the subjacent motivation [5], and its predictor variables to more efficiently prevent aggressive and violent behavior.

Reactive aggression is a reaction to a provocation or a threat (real or imagined) [6]. Moreover, it relates to low cognitive planning, impulsiveness or intense emotional activation, and emotional instability [7]. This type of aggression could be explained by the frustration–aggression model, which postulates that aggression results as a hostile reaction to frustration [8]. However, proactive or instrumental aggression is characterized by emotional detachment and emotional callousness. It is about an instrumental aggression related to a way of acting in which ‘the end justifies the means’. It is carried out to solve conflicts or to obtain benefits or reinforcement (such as power or domination) [9], typical of bullying, which has been described as a proactive type of aggression [10]. Proactive aggression may be explained by the social learning theory [9].

The encouraged traditional masculinity favors the development of aggressive behavior. In fact, gender stereotypes have an influence on intimate partner violence [11], gender violence [12], bullying [13,14], and cyber aggression [15]. The role of the male gender includes norms that encourage aggressive behavior and the traditional feminine role emphasizes behaviors considered protective from aggression [16]. In fact, femininity was negatively related to adolescent Chinese boys externalizing problem behaviors [17].

Traditional masculinity is considered as the negative and socially aversive traits and behaviors related to idealized societal masculine norms, and is characterized by instrumental personality traits such as aggression, self-affirmation, social dominance, and a lack of consideration for others [18,19,20,21]. Everybody, including both boys and girls, expresses behaviors and attitudes consistent with traditional masculinity because the values, traits, and behaviors are not typical of a specific gender [22]. In the last few decades, the social changes have contributed to the transformation of the prototypical gender functions and to the blurriness of the content of the stereotypes [23]. This way, the meaning of masculinity and femininity, as social fenomena, could have changed. However, boys tend to display attributes consistent with traditional masculinity [24,25], while girls tend to display attributes consistent with femininity (such as caring, being more sensitive to the needs of others, understanding, compassion, warmth, affection, tenderness, crying easily, submissiveness...) [26] as a consequence of a sexist socialization. Therefore, some research highlights that boys and girls continue to manifest the same gender stereotypes as in the past [27].

Cognitive social theory [28,29,30,31] states that boys and girls acquire beliefs about what is the appropriate behavior according to their gender from social influences such as family, peers, the media, their culture, etc. Furthermore, adolescents watch the asymmetrical power relations around them through either real or symbolic models. These models are then internalized in childhood and adolescence. The need for boys and girls to support a stable image of themselves encourages them to consolidate gender stereotypes [32]. In this way, the social norms for gender become rooted belief systems or cognitive scripts which regulate and guide their behavior [30].

Furthermore, researchers suggest that gender socialization is related to emotion regulation [33]. For example, traditional masculinity has limited the emotional expression and disposition to respond to or acknowledge feelings [34]. However, most studies on aggression have only focused on analyzing differences between boys and girls, where the boys have obtained higher scores in reactive and proactive aggression [35] and the relation between masculinity and reactive and proactive aggression has not been greatly studied, making the results of these studies contradictory [36,37]. While in Oransky [36] endorsement of traditional male role norms showed a negative relationship with reactive aggression and a positive relationship with proactive aggression, in Sánchez and Moreira y Mirón [37], masculinity, in both men and women, had a positive association with reactive aggression, in complete opposition to Oransky’s study, while femininity inhibited this kind of aggression among women. Nevertheless, the masculinity dimension has a positive and significant to proactive aggression.

In addition, adolescents who are able to manage negative effects through emotional regulation have more personal resources that protect them from maladaptive behaviors, such as aggression [38]. Emotional regulation is understood as the effort the person makes to change the course of their emotional expression through cognitive and expressive processes [39]. The lack of emotional regulation, such as a poor impulse control, has been related to aggression [40,41], and in particular with reactive and proactive aggression [42,43,44].

Moreover, emotional regulation has been related to regulatory emotional self-efficacy [45]. The literature points out that behavior relates to the beliefs that people have of their own abilities in a particular situation, as this will influence motivation and the effort made [46]. The concept of regulatory emotional self-efficacy in managing negative effects was postulated by Caprara [47], and it was based on the self-efficacy theory [48].

**Figure 1:**
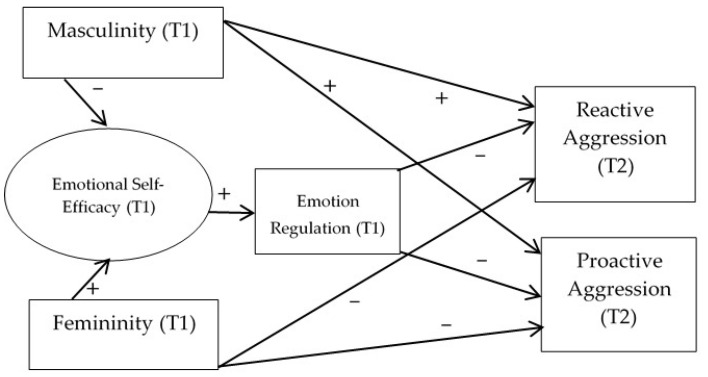
Starting theoretical model (+ = positive relationship; − = negative relationship). T1 = Time 1; T2 = Time 2. Emotional Self-Efficacy = Regulatory Emotional Self-Efficacy.

Therefore, the objective of this study is to examine the role of gender-related traits (masculinity/femininity) in reactive and proactive aggression, through regulatory emotional self-efficacy and emotion regulation, through a longitudinal study, with Valencian adolescents between 12 and 15 years of age [33,36,37,49,50]. We focus on early adolescence [51] as this stage gathers specific characteristics and physiological and emotional changes, the search for a differentiated identity as well as the beginning of intimate relationships with parents, and the centrality importance of the body. Therefore, conforming to social norms becomes paramount which will give gender stereotype a relevant weight as they are referents and models for the construction of identities, generating expectations and attitudes that maintain the social system [52]. We postulate that the self-attribution of traditional masculine and feminine traits will relate to reactive and proactive aggression through emotional self-efficacy, thus being a negative relation between masculinity and emotional self-efficacy and a positive relation between femininity and self-efficacy. Likewise, emotional regulation and reactive and proactive aggression will relate negatively. Therefore, emotional self-efficacy and emotional regulation will be mediator variables (Hypothesis 1). Gender will moderate this mediation (Hypothesis 2). The relationship between emotional self-efficacy and emotional regulation will be positive (Hypothesis 3). Furthermore, reactive and proactive aggression will relate in a positive and direct way to masculinity (Hypothesis 4), and in a negative way to femininity (Hypothesis 5). Therefore, masculinity could act as a vulnerability factor of reactive and proactive aggression and femininity, emotional self-efficacy, and emotional regulation could act as protective factors of the reactive and proactive aggression (Figure 1). The following model was tested:

## 2. Materials and Methods

### 2.1. Participants

This research monitored participating adolescents for two years in a longitudinal study in Valencia, Spain. A total of 390 adolescents (208 boys and 182 girls), aged 12–15 years (Mage = 12.74 years, SDage = 0.75), fully completed both surveys and comprised the final sample. In the first wave, adolescents were either in the first year of secondary school. Participating schools were randomly selected from the list of all schools in Valencia with students enrolled in compulsory secondary education (educación secundaria obligatoria—ESO). In total, 9 schools participated in the study.

In addition, 74.1% of adolescents came from two-parent households, and 20.2% of the parents were divorced. Regarding the educational level, 19.7% of mothers had less than a secondary school diploma, 33.2% had a secondary school diploma or equivalent, and 47.1% had some university education. Furthermore, 25.4% of fathers had less than a high school diploma, 35.5% had a high school diploma or equivalent, and 39.1% had some university education. Additionally, 91.4% of students reported being from Spain. Small percentages of the remaining adolescents self-identified themselves as being from Latin America and Eastern European countries.

### 2.2. Research Procedure

The assessment was carried out by trained researchers in the classroom in 50-min sessions during school hours. Participation by adolescents was voluntary and adolescents were free to decline to participate. The study was approved by the Valencian Government. Parental consent and approval from the School Council were obtained. The research followed all ethical guidelines included in the Helsinki Declaration under current regulations, respecting respondents’ anonymity for both data collection and data analysis. This research had a favorable response from the University Ethics Committee because it is required for the concession of Research Projects.

### 2.3. Measures

Reactive and Proactive Aggression were assessed with the ReactiveProactive Aggression Questionnaire (RPQ) [7]. RPQ is a self-report instrument consisting of 23 items that reflect reactive or proactive aggression. Participants are asked to rate each item as to its frequency of occurrence on a scale from 0 (never) to 2 (often), with higher scores representing greater aggression. The RPQ has demonstrated adequate reliability and validity properties when used with child and adolescent samples [7]. With the current sample, the RPQ demonstrated good reliability for both the reactive (11 items; α = 0.84, at time 2) and proactive aggression items (12 items; α = 0.93, at time 2). The total score of each scale is the mean of the items score.

Traditional Masculinity and Femininity were assessed with the Sexual Roles Inventory (BSRI—Short version) [53,54]. This questionnaire was used to find out the degree in which people accept gender stereotypes asking participants to indicate the degree to which they believe each characteristic describes them. It measures 2 scales of 18 items each: one scale assesses instrumentality (traits associated to traditional masculinity) and the other scale assesses expressivity (traits associated to traditional femininity). The instrument assesses attributes which have traditionally been associated to masculinity and femininity. Gender is a dynamic construct, whereby masculine and feminine are defined and interpreted in different ways and in different cultures (even inside the same social organization people give them different meanings). Therefore, it is difficult to specify what masculinity and femininity are. In our study, we have taken into account attributes related to instrumentality and expressivity. The instrumentality has been related to masculinity and expressivity to femininity. Example items include instrumentality “Athletic”; and expressivity “Caring”. With the current sample, the BSRI demonstrated good reliability for both masculinity (18 items; α = 0.79, at time 1) and femininity (18 items; α = 0.78 at time 1). The total score of each scale is the mean of the items score. The fit statistics have been obtained for the questionnaire of the factors analyzed. They are as follows: masculinity *χ^2^* (36) = 64.34, *p* = 0.000; Bentler comparative fit index, CFI = 0.93; Standardized root mean residual, SRMR = 0.05; and femininity *χ*^2^ (36) = 71.28, *p* = 0.000; CFI = 0.93; SRMR = 0.04. 

Regulatory emotional self-efficacy was assessed with the Regulatory Emotional Self-Efficacy Scale [55]. This instrument evaluates self-efficacy beliefs in the domain of emotion regulation, using a five-point Likert scale ranging from be unable (1) to fully capable (5). Two subscales assessed by nine items were included in this study: (a) perceived self-efficacy in managing anger/irritation assessed the perceived ability to manage negative affect in the face of anxiety-arousing threats, anger provocation, rejection, and disrespect, and to control worrisome ruminations when things go wrong (“I can Manage negative feelings when reprimanded by my parents or significant others”, α = 0.78 at time 1); and (b) perceived self-efficacy in managing despondency/distress measured the perceived ability to manage negative affect in the face of despondency and discouragement (“I can keep from getting discouraged in the face of difficulties”, α = 0.71 at time 1). The total score of each scale is the mean of the items score. The fit statistics were obtained for the latent variable analyzed. They are as follows: regulatory emotional self-efficacy *χ^2^* (24) = 41.17, *p* = 0.01; CFI = 0.97; SRMR = 0.04.

Emotions regulation was assessed with the Resilience Appraisals Scale (RAS) [56]. This measures coping or emotional regulation when facing stressful life events. It is made of 4 items based on the dimensions of emotional regulation. It is assessed using a five-point scale ranging from completely disagree to completely agree. Example items “I can manage my emotions”; “I can contain my unpleasant emotions”. With the current sample, the RAS demonstrated good reliability for emotion regulation (α = 0.85, at time 1). The total score of the scale is the mean of the items score.

### 2.4. Statistical Procedure

SPSS 26 (IBM Corp. Armonk, NY, USA) was used to calculate descriptive analysis and to perform correlation analysis to carry out to test the relations among variables. In addition, structural equations modelling (SEM) in Mplus 6.1 (Muthén & Muthén, Los Angeles, CA, USA) [57] was employed to explore a two-wave longitudinal model. The following goodness-of-fit indices were used: chi-square divided by degrees of freedom (χ^2^/d.f.) and Bentler comparative fit index (CFI). Standardized root mean residual (SRMR) was used to measure error [58]. Furthermore, indirect effects were tested using the bias corrected bootstrap confidence interval method in Mplus (Muthén & Muthén, Los Angeles, CA, USA) [59,60]. Next, multi-group analyses were conducted to examine if the patterns of associations differed based on gender. A chi-square difference test was conducted to examine significant change in the chi-square statistic for the constrained model [57].

## 3. Results

### 3.1. Descriptive Statistics, and Correlational Analyses

Means, standard deviations, means according to gender, and results for the correlations among the variables are presented in Table 1. To ascertain the differences among the participants by gender, we performed a one-factor analysis of variance (ANOVA) with the studied variables. The ANOVAs confirmed significant differences in proactive aggression (*F* = 6.687, *p* = 0.01), emotion regulation (*F* = 8.44, *p* = 0.004), regulatory emotional self-efficacy (anger) (*F* = 13.03, *p* = 0.000), regulatory emotional self-efficacy (distress) (*F* = 13.05, *p* = 0.000), masculinity (*F* = 7.21, *p* = 0.008), and femininity (*F* = 13.85, *p* = 0.000). The correlations showed that reactive aggression is significantly negatively correlated to emotion regulation and regulatory emotional self-efficacy (perceived self-efficacy in managing anger/irritation and perceived self-efficacy in managing despondency/distress), and femininity. Furthermore, reactive aggression is significantly positively related to masculinity.

In addition, proactive aggression is significantly negatively correlated to femininity and significantly positively correlated to masculinity. Finally, reactive and proactive aggression are significantly positively correlated to each other (Table 1).

### 3.2. Structural Equation Model

**Figure 2:**
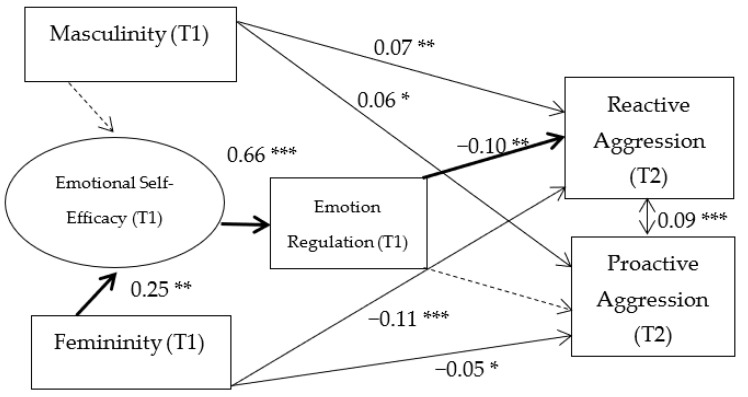
Path model of the relations among reactive aggression, proactive aggression, emotion regulation, regulatory emotional self-efficacy, masculinity, and femininity across youth from Spain. Standardized coefficients are depicted. T1 = Time 1; T2 = Time 2. Emotional self-efficacy = regulatory emotional self-efficacy. Normal arrows depict significant relationships. Interrupted arrows depict non-significant relationships. Indirect effects are depicted in black. * *p* < 0.05; ** *p* < 0.01; *** *p* < 0.001.

The longitudinal model was analyzed using structural equations modeling. The model captured the relation between latent and observed variables in two times. The latent variable was regulatory emotional self-efficacy, created by perceived self-efficacy in managing anger/irritation and perceived self-efficacy in managing despondency/distress. The observed variables were reactive aggression, proactive aggression, emotion regulation, masculinity, and femininity. The model captured the relationships between masculinity (time 1), femininity (time 1), and reactive and proactive aggression (time 2) through regulatory emotional self-efficacy (time 1) (perceived self-efficacy in managing anger/irritation and perceived self-efficacy in managing despondency/distress), and emotion regulation (time 1). The direct relationship between regulatory emotional self-efficacy and emotion regulation was also studied, and the relationship of masculinity/femininity and regulatory emotional self-efficacy. Furthermore, the relationship between emotion regulation and reactive/proactive aggression was also considered. The results indicate a good fit between the model and the empirical data: *χ*^2^ (25) = 448.120, *p* = 0.000. The following fit indices were also obtained: CFI = 0.94, showing a very good fit. Finally, standardized root mean square residual (SRMR) = 0.037. Values below 0.10 indicate acceptable error and values around 0.06 indicate a very good fit [8]. Figure 2 shows the path values (Hypothesis 3–5).

Bias corrected bootstrap confidence intervals suggested that there was a significant indirect effect from femininity to reactive aggression via regulatory emotional self-efficacy and emotion regulation ((*β* = −0.04, *p* = 0.02) (Table 2) (Hypothesis 1). In testing moderation by gender, the unconstrained model [*χ*^2^ (10) = 36.48] and the constrained model [*χ*^2^ (24) = 18.96] were not significantly different as determined by a chi-square difference test [*χ*^2^ (14) = 17.43, *p* = 0.23], suggesting no moderation by gender (Hypothesis 2).

## 4. Discussion

The main objective of this study was to examine the role of gender-related traits (masculinity-femininity) in reactive and proactive aggression, through regulatory emotional self-efficacy and emotion regulation.

We postulated that the self-attribution of masculine and feminine traits in Time 1 will relate to reactive and proactive aggression in Time 2, through emotional self-efficacy and emotional regulation from Time 1. The results partially support this hypothesis as femininity relates to reactive aggression through regulatory emotional self-efficacy and emotion regulation. The characteristics related to femininity, as well as the evaluated emotional processes, emotional self-efficacy, and emotional regulation, become protective factors of reactive aggression. Our study is in line with other studies which point out that reactive aggression relates to a lack of emotional regulation [7,42], and the latter one, at the same time, relates to gender socialization [33]. Nevertheless, in our study, obtaining high scores in masculinity has not been related to the emotional processes. Ours results lean partially on another study which shows that men who strongly adhere to the masculine gender norms and had an inability to control impulses may produce aggressive behavior toward perceived gay men [50]. Likewise, emotional regulation self-efficacy has also been a mediator variable between femininity and emotional regulation. These results lean on the study carried out by Hadley, Mowbray, and Jacobs [49], which demonstrated that self-efficacy about aggression was a mediator variable between approval of aggression and proactive aggression. However, our study implicates reactive aggression, not proactive aggression. In the same way, both emotional self-efficacy and emotional regulation have mediated the relation between femininity and reactive aggression, reinforcing the results of other studies which show that having more personal resources protect adolescents form aggression [38].

Emotional self-efficacy and emotional regulation did not mediate the relation between femininity and proactive aggression, which has related directly with masculinity. This could be due to proactive aggression being an instrumental aggression to solve conflicts or obtain benefits or reinforcements, such as domination or power [9], and, for this reason, it does not relate to emotional regulation. In spite of the adolescent being able to regulate their emotions very well, if they have learned to get what they want showing aggression and violence, emotional regulation will not have a key role in this process [61]. Proactive aggression does not appear due to a low capacity to regulate frustration, as is the case with reactive aggression. In bullying, proactive aggression behavior is carried out to achieve power or domination [10]. Likewise, proactive aggression has similarities to dating violence and gender violence, as these kinds of violence mean an imbalance or abuse of power [62], and it is the beliefs of the person which motivate these kinds of violence. Regarding hypothesis 2, which stated that gender would moderate the mediator role of the regulatory emotional self-efficacy and emotion regulations variables, it has been impossible to confirm, as the results obtained in the multi-group model by gender have not been significant, which would be in line with other studies that stated that both boys and girls express behaviors consistent with traditional masculinity [22], and, therefore, the consequences related to emotional regulation and aggression will depend on the self-attribution of masculine and feminine traits.

As for the third hypothesis, in which it was expected that the relationship between emotional self-efficacy and emotional regulation was a positive one, the results sustain this hypothesis as there is a positive and significant relation between these two variables [46]. As we mention in the theoretical introduction, the beliefs we have about our own abilities will influence the effort we make to attain something and, therefore, will modulate our behavior. In this case, if we think that we have the abilities to regulate our emotions, like anger and frustration, we will regulate our emotions in a more efficient way.

Furthermore, the fourth and fifth hypothesis, which stated that reactive and proactive aggression will relate directly and positively to masculinity and negatively to femininity, are also confirmed after our study. Therefore, masculinity could act as a vulnerability factor of reactive and proactive aggression, and femininity could act as a protective factor of reactive and proactive aggression. In hypothesis 4, our results support the hypothesis that masculinity would relate to reactive and proactive aggression. These findings are consistent with the idea that the self-attribution of masculine traits, considered as personality characteristics, may lead adolescents to endorse reactive and proactive aggression. The relationship between masculinity and reactive and proactive aggression may be explained in terms of cultural representations, and social expectations. Cultural representations are internalized through social expectations, norms, roles, values, beliefs, attitudes, and behaviors, which establish categories that cognitively affect the perceptions and attributions made by people both on themselves and on other people [30,31]. Therefore, gender stereotypes might be related to self-concept and the direction and management of one’s own actions [63]. Traditional masculinity is characterized by instrumental personality traits such as aggression, self-affirmation, social dominance, and lack of consideration for others [18,19,20,21], and these traits are internalized in childhood and adolescence. The traditional masculine role includes norms that encourage many of the behaviors considered aggressive [16,37]. Likewise, the traditional feminine role emphasizes aggression very little [16], which would protect them from carrying out aggressive behaviors [17]. In fact, traditional femininity, which encompasses attributes related to emotional expressivity and the attention to other has been related directly to less reactive and proactive aggression, in this way, acts as a protective factor for aggressive behaviors.

Before stating the strengths of this study, it is necessary to comment on its limitations. First of all, all variables were obtained using self-reporting data. Even so, it has been demonstrated that the self-informed information of the adolescents is more reliable and has greater predictive value than that obtained from the families [64]. Despite this, for future studies, it would be interesting to take into consideration other sources of information to corroborate the reliability of the evaluated source. Likewise, even though it is a longitudinal study, it only contemplates to assessment times. In future studies, it would be advisable to contemplate more assessment times. The longitudinal study brings a strength due to its rigor in investigation, especially in adolescence, when the person is developing and important changes take place in different cognitive, emotional, and behavioral processes. Furthermore, it would be interesting to know what happens in middle and late adolescence. Additionally, we have used a quantitative methodology. In future studies, it would be interesting to use a qualitative methodology to complement the quantitative one, above all, to assess the constructs of masculinity and femininity which are in constant change [65]. Lastly, another limitation is that the questionnaire used to evaluate masculinity and femininity assesses variables traditionally related to these constructs but, being social constructs, they are dynamic and are changing constantly and at present it might not necessarily reflect participants’ “masculine” and “feminine” traits.

As for the strengths of this study, it should be noted that, in our model, the assimilation of gender stereotypes; traditional masculinity and femininity; and emotional processes, emotional self-efficacy, and emotional regulation have been taken into account as well as their relationship with reactive and proactive aggression of the year after. No other studies that incorporate these variables in the same model and in a longitudinal study are known prior to the carrying out of the present study. The main findings support the need to build a model of relationship between boys and girls, eradicating a traditional model of relationship based on traditional gender roles to eradicate reactive and proactive aggression behavior [66]. Moreover, the importance of working the emotions and emotional management is confirmed, in particular to diminish reactive aggression. The characteristics associated to traditional femininity encourage emotional self-efficacy and emotional regulation. The implications of our study are to contribute data to support the need to educate in equality, prosocial values, empathy, and emotional regulation for all genders and not to educate in attributes and values traditionally associated to the traditional and toxic masculinity which generates aggression and violence. Following studies by Rosen and Nofziger [14] and Ray and Parkhill [50], violence prevention programs should incorporate women and men role models which break down traditional gender stereotypes and promote acceptance of gender diversity, and the values traditionally associated to femininity. Furthermore, the violence prevention programs could offer adolescents information on gender stereotypes, and traditional masculinity, as it pertains to aggression and violence. In addition, adolescents could acquire strategies to manage their emotions that may be used in place of aggression behaviors and violence. The implications call for future research and interventions attentive to breaking down traditional gender stereotypes and improved emotion regulation skills in adolescents. Such lessons would help to foster healthier relationships in life, and may be very helpful to reduce aggressive behaviors and violence.

## 5. Conclusions

Femininity relates to reactive aggression through regulatory emotional self-efficacy and emotion regulation. Both regulatory emotional self-efficacy and emotion regulation have mediated the relationship between femininity and reactive aggression. The attributes related to femininity, as well as the emotional processes studied, emotional self-efficacy, and emotional regulation, become protective factors for reactive aggression. In the same way, the relationship between emotional self-efficacy and emotional regulation has been positive. Lastly, traditional masculinity relates in a positive and direct way to reactive aggression, acting as a vulnerability factor of the aggression behaviors studied, and femininity relates in a negative and direct way to reactive and proactive aggression, acting as a protective factor of the aggression behaviors studied.

## Figures and Tables

**Table 1:** Descriptives and correlation matrix for emotion regulation (T1), regulatory emotional self-efficacy (T1), masculinity (T1), femininity (T1), reactive aggression (T2), and proactive aggression (T2).

Variable	1	2	3	4	5	6	7
Reactive Aggression (T2)	-						
Proactive Aggression (T2)	0.63 **	-					
Emotion Regulation (T1)	−0.19 **	−0.03	-				
Regulatory Emotional Self-Efficacy (anger, T1)	−0.22 **	−0.01	0.62 **	-			
Regulatory Emotional Self-Efficacy (distress, T1)	−0.11 *	0.04	0.50 **	0.58 **	-		
Masculinity (T1)	0.15 **	0.15 **	0.01	0.03	0.08	-	
Femininity (T1)	−0.19 **	−0.16 **	0.01	0.10 *	−0.02	0.14 **	-
Mean	1.64	1.29	3.47	3.01	3.33	3.98	4.82
SD	0.38	0.39	0.88	0.94	0.79	1.01	0.88
Gender							
Boys	1.65	1.34	3.59	3.17	3.47	4.11	4.67
Girls	1.63	1.24	3.33	2.83	3.18	3.84	4.99

Note: * *p* < 0.05; ** *p* < 0.01; regulatory emotional self-efficacy (anger) = perceived self-efficacy in managing anger/irritation; regulatory emotional self-efficacy (distress) = perceived self-efficacy in managing despondency/distress; SD = standard deviation; 1 = reactive aggression (T2); 2 = proactive aggression (T2); 3 = emotion regulation (T1); 4 = regulatory emotional self-efficacy (anger, T1); 5 = regulatory emotional self-efficacy (distress, T1); 6 = masculinity (T1); and, 7 = femininity (T1).

**Table 2:** Indirect effects.

Variables	*β*	*p*
Masculinity → Emotional Self-Efficacy → Emotion Regulation → Reactive Aggression	−0.002	0.89
Femininity → Emotional Self-Efficacy → Emotion Regulation → Reactive Aggression	−0.04	0.02
Masculinity → Emotional Self-Efficacy → Emotion Regulation → Proactive Aggression	0.000	0.93
Femininity → Emotional Self-Efficacy → Emotion Regulation → Proactive Aggression	−0.01	0.37

Note: → = it depicts direct effects between variables.

## Data Availability

The data presented in this study are available on request from the corresponding author. The data are not publicly available due to are data of a Research Project of Valencian Government.
